# A Control Chart Based on Cluster-Regression Adjustment for Retrospective Monitoring of Individual Characteristics

**DOI:** 10.1371/journal.pone.0125835

**Published:** 2015-04-29

**Authors:** Hong Choon Ong, Ekele Alih

**Affiliations:** School of Mathematical Sciences, Universiti Sains Malaysia, 11800 Pulau Pinang, Malaysia; Universidad de Valladolid, SPAIN

## Abstract

The tendency for experimental and industrial variables to include a certain proportion of outliers has become a rule rather than an exception. These clusters of outliers, if left undetected, have the capability to distort the mean and the covariance matrix of the Hotelling’s *T* ^2^ multivariate control charts constructed to monitor individual quality characteristics. The effect of this distortion is that the control chart constructed from it becomes unreliable as it exhibits masking and swamping, a phenomenon in which an out-of-control process is erroneously declared as an in-control process or an in-control process is erroneously declared as out-of-control process. To handle these problems, this article proposes a control chart that is based on cluster-regression adjustment for retrospective monitoring of individual quality characteristics in a multivariate setting. The performance of the proposed method is investigated through Monte Carlo simulation experiments and historical datasets. Results obtained indicate that the proposed method is an improvement over the state-of-art methods in terms of outlier detection as well as keeping masking and swamping rate under control.

## Introduction

Following Staudte and Sheather [1], an outlier can be defined as an observation that is far from the bulk of the data. Similarly, Hampel et al. [2] defined outliers to be observations which deviate from the pattern set by majority of the data. Since this outlying observations depart from the general trend dictated by the bulk of the data, its identification in the dataset plays an important role in many practical applications. The dataset may contain outliers with abnormal values that are arbitrarily larger or smaller than the normal observations. This could lead to misjudgment of analysis in areas such as regression analysis, analysis of variance, statistical process control (*SPC*) and profile monitoring. Therefore, outlier identification is an important task prior to data analysis especially in monitoring product quality of a production process. Historical data often suffer from transcription errors and problems in data quality. These errors make historical data prone to outliers.

According to Montgomery et al. [3], outliers are identified as observations that produce residuals that are considerably larger in absolute values than the others; say, three or four sigma from the mean. This idea can be applied in *SPC*. Consider a situation where the objective of a multivariate control chart is to detect the presence of assignable causes of variation in multivariate quality characteristics. In particular, for a retrospective phase I analysis of a historical dataset, the objective is two fold: (i) to identify shifts in the mean vector that might distort the estimation of the in-control mean vector and covariance matrix; (ii) to identify and eliminate multivariate outliers. The purpose of seeking an in-control subset of historical dataset is to estimate in-control parameters for use in a phase II analysis. To achieve these objectives, individual retrospective multivariate control charts are constructed to determine if in a multivariate sense, the already obtained, sequentially ordered data points **X** = {**x**_*ij*_}_*i* = 1, ⋯, *n*, *j* = 1, ⋯, *p*_ ⊂ ℝ^*p*^ are stable, that is, free of outliers, upsets or shifts. Fan et al. [4] refer to this kind of analysis as a retrospective Phase I analysis of a historical data set (*HDS*). The sample mean vector and covariance matrix are estimated from **X**. From these estimates, Hotelling’s *T* ^2^ chart is constructed and used to flag outliers and mean shifts in the process.

According to Marcus and Pokojovy [5], given the multivariate quality characteristic, **X**, the Hotelling’s *T* ^2^ statistic computed for {**x**_*ij*_}_*i* = 1, ⋯, *n*, *j* = 1, ⋯, *p*_ ⊂ ℝ^*p*^ at time *i* is

$$\begin{array}{c} T_{i}^{2}={\left(x_{i}-m_{x}\right)}^{\prime }{\hat{\Sigma }}^{-1}\left(x_{i}-m_{x}\right) \end{array}$$ (1)

where **m**_**x**_ is the sample mean given by

$$\begin{array}{c} m_{x}=\frac{1}{n}\sum_{1=1}^{m}x_{i} \end{array}$$ (2)

and $\hat{\text{Σ}}$ is the sample covariance matrix given by

$$\begin{array}{c} \hat{\Sigma }=\frac{1}{n-1}\sum_{i=1}^{m}\left(x_{i}-m_{x}\right){\left(x_{i}-m_{x}\right)}^{\prime }. \end{array}$$ (3)

If $T_{i}^{2}$ exceeds the upper control limit (*UCL*) defined as

$$\begin{array}{c} UCL=\frac{{\left(n-1\right)}^{2}}{n}Beta_{\alpha ;\frac{p}{2},\frac{\left(n-p-1\right)}{2}}, \end{array}$$ (4)

then **x**_*i*_ is declared as an outlier or a special cause (out-of-control) is assumed to have occurred at sample number *i* or before it. Eq (4) assumes the **x**_*i*_’s are independent and come from a multivariate normal distribution. However, the *T* ^2^ are correlated because they each depend on the same **m**_**x**_ and $\hat{\text{Σ}}$ (Mansion and Young [6]).

The classical methods for detecting mean shift and outliers such as the one described in Eq (1) are powerful when the dataset contain only one outlier. Nevertheless, the power of these techniques reduces significantly when more than one outlying observations are present in the datasets. This loss of power is typically due to what can be referred to as the *masking* and *swamping* problems. Furthermore, these techniques do not always succeed in identifying mean shift and spurious outliers because they are based on several assumptions which are rarely met. Thus, they are affected by the observations that they are supposed to detect. Therefore, a technique devoid of these problems is desirable.

Let $D_{i}({\text{m}}_{\text{x}},\hat{\text{Σ}}{\text{m}}_{\text{x}})=f({\text{x}}_{i}-{\text{m}}_{\text{x}},\hat{\text{Σ}}),\;i=1,\cdots ,n$ be a suitable metric for measuring the distance between the *i* ^*th*^ observation **x**_*i*_ and a location (mean) estimator **m**_**x**_ in Eq (2), relative to a measure of dispersion (covariance matrix), $\hat{\text{Σ}}$ in Eq (3). Several forms of **m**_**x**_ and $\hat{\text{Σ}}$ have been discussed in literature (see for instance, Rousseeuw [7], Rousseeuw and van Driessen [8], Billor et al. [9] and Hubert et al. [10]). The most frequently used form of $f({\text{x}}_{i}-{\text{m}}_{\text{x}},\hat{\text{Σ}}),\;i=1,\cdots ,n$ is defined in Eq (1). The classical alternatives for **m**_**x**_ and $\hat{\text{Σ}}$ are defined in Eqs (2) and (3) respectively. Eq (1) can be referred to as the squared Mahalanobis distance. It will be referred to as $T_{i}^{2}$ for simplicity.

A large value of $T_{i}^{2}$ is a likely indication that the observation with indices corresponding to it is an outlier or better still, an out-of-control process. However, two problems occur in reality. First, outliers or out-of-control process may not essentially have large values of $T_{i}^{2}$. For instance, a small cluster of outliers will attract **m**_**x**_ and will inflate $\hat{\text{Σ}}$ in its direction, leading to small values for $T_{i}^{2}$. This problem is referred to as the *masking* problem since the presence of one outlier masks the appearance of another outlier.

Secondly, not all observations with indices corresponding to large $T_{i}^{2}$ values are essentially outliers or an out-of-control process. For instance, a small cluster of outliers will attract **m**_**x**_ and inflate $\hat{\text{Σ}}$ in its direction and away from some other observations which belong to the trend suggested by the bulk of datasets, thereby yielding large $T_{i}^{2}$ values for these observations. This problem is referred to as *swamping* problem.

Masking and swamping phenomena occur because **m**_**x**_ and $\hat{\text{Σ}}$ are not robust. One way to remedy these problems is to use more robust estimators for Eqs (2) and (3) as an alternative to the location and covariance matrix. The resulting $T_{i}^{2}$ could be used to effectively detect outliers or sustained shifts in the mean vector of the quality characteristics.

Consider the minimum volume ellipsoid, (*MVE*) of Rousseeuw [7] which covers at least half of the observations to construct robust estimators for location and dispersion in Eqs (2) and (3). The mean vector and covariance matrix of the observations included in the *MVE* are robust location and dispersion matrix estimators. The advantage of *MVE* estimators for location and dispersion is that they have a high break down point, (*BDP*) of approximately 50% (Lopuhaa and Rousseeuw [11]). They can also resist the presence of substantial amount of outlier in dataset as well as being able to effectively detect sustained shifts in the mean vector of the quality characteristics. However, the *MVE* is computationally intensive, and it may not even be computationally feasible, to implement the *MVE* algorithm estimators. As an illustration of the *MVE*’s computational infeasibility, for an *n* × *p* data matrix **X**, if *h* is the integer part of $\frac{\left(n+1\right)}{2}$, then the algorithm needs to compute the volumes of $\frac{n!}{h!(n-h)!}$ ellipsoids and to choose the ellipsoid with the minimum volume. This means that in an instance where *n* = 20, there are 184,756 such ellipsoids, for *n* = 25, there are 5,200,300 such ellipsoid and for *n* = 30, there are over 155 million ellipsoids to compute and to select the ellipsoid with the minimum volume. This enormous computational demand of *MVE* which grow geometrically as the sample size, *n* increases is common to nearly all methods that implement elemental sets or resampling algorithm.

Another robust estimator of mean vector and covariance matrix that can accommodate up to 50% outliers in dataset and also work well for Eqs (2) and (3) in computing $T_{i}^{2}$ is the minimum covariance determinant estimator, (*MCD*). Rousseeuw and van Driessien [8] proposed a fast algorithm for computing the the *MCD* estimator and they referred to it as *FastMCD*. The *FastMCD* correspond to finding the *h* points for which the classical tolerance ellipsoid has minimum volume, and then taking it center. Consider all $C_{h}^{n}$ subsets, and compute the determinant of the covariance matrix for each subset. The subset with the smallest determinant is used to calculate the usual 1 × *p* mean vector, **m** and corresponding *p* × *p* covariance matrix, $\hat{\text{Σ}}$. This estimation procedure describes the *MCD* algorithm. The *MCD* estimator for location and dispersion are actually maximum likelihood estimators when *h* observations are from the multivariate normal distribution, while the other (*n* − *h*) observations are from a different, mean shifted, multivariate normal distribution. Like the *MVE*, it is equivariance and has high breakdown value of 50%. Cator and Lopuhaa [12] examined the asymptotic expansion of *MCD* estimator and stated that the *MCD* estimator is efficient and root $\sqrt{n}$-consistent. Furthermore, Cator and Lopuhaa [13] derived the influence function for the *MCD* estimator. The *MCD* estimator is robust and equivariant and can resist the presence of substantial amount of outliers in dataset as well as being able to effectively detect sustained shifts in the mean vector of the quality characteristics. However, it often biasedly estimate the center (mean vector) and as a result underestimates the covariance matrix. According to Pison et al. [14], the *MCD* estimator is inconsistent and underestimates the volume of the covariance matrix such that robust distances constructed from it are too large resulting in identifying too many observations as outliers when they are originally inliers. This phenomenon is called swamping effect. Swamping makes the control chart identifies too many observations as outlying thereby declaring an originally in-control-process as out-of-control process

Billor et al. [9] proposed forward search algorithm called *BACON* with two versions of the initial subsample. The algorithm has two “*initial starts*” in which version one uses the non-robust but equivariant classical mean vector while version two uses the robust but not equivariance median. The *BACON* algorithm is computable, easy to implement and can resist the presence of substantial amount of outlier in dataset as well as being able to effectively detect sustained shifts in the mean vector of the quality characteristics. However, the version one of *BACON* is not robust but equivariance. Hence, the subsequent iterations may not be robust as it will depend on the robustness of the initial subset. The version two of *BACON* is robust but not equivariance and hence the subsequent iterations may not be equivariant.

Hubert et al. [10] proposed the deterministic minimum covariance determinant (*DetMCD*) algorithm for estimating the location and dispersion matrix in Eqs (2) and (3) when datasets contains outliers and are presumed to exhibit drift or a mean shift in a process. The *DetMCD* uses six different initial estimators to select six initial subsets denoted as $h_{0}=\lceil \frac{n}{2}\rceil$. From the six initial *h*_0_, a Mahalanobis type distance measure *d*_*i*, *l*_, (*i* = 1, ⋯, *n*; *l* = 1, ⋯,6) is computed. Thereafter, the method selects for all six initial estimates, the *h* observations **x**_*i*_ with the smallest *d*_*i*, *l*_ and apply the concentration steps (*C*-steps) until convergence.

According to Hubert et al. [10], the *DetMCD* estimator is highly robust and near equivariant. Thus, it can resist the presence of substantial amount of outliers in dataset as well as being able to effectively detect sustained shifts in the mean vector of the quality characteristics. However, the computational requirement of *DetMCD* is highly enormous. Imagine the computational rigor in computing six different initial estimates that are independent, and can each be seen as “stand alone” estimator. The accompanying *C*-step also requires enormous computation to converge. Moreover, since the *DetMCD* use the *FastMCD* objective, it will inherit the ills of *FastMCD* as noted in Pison et al. [14]. Thus, it often biasedly estimate the center and as a result underestimates the dispersion matrix. It is inconsistent and underestimates the volume of the covariance matrix such that robust distances constructed from it are too large and hence identifying too many observations as outliers when they are originally inliers, leading to swamping effect.

Vargas [15] and Jensen et al. [16] studied the performance and evaluated several different retrospective multivariate control charts methods whose classical mean vector and covariance matrix in Eqs (2) and (3) are constructed from robust estimation algorithms. Each of the charts were based on a *T* ^2^ statistic calculated using a selected combination of robust mean vector and covariance matrix estimators. Jobe and Pokejovy [5] noted that most of the robust estimators considered (particularly the *MVE* and *MCD*) do not take time (*i*) into consideration. This is a drawback in relation to detecting certain outlier configurations such as sustained shifts in the mean vector (see Jobe and Pokojovy [5] for details). The inability of some robust methods to account for time *i* in constructing the $T_{i}^{2}$ may be linked to non equivariance tendency. Some methods that work well in this scenario, accounting for time includes that of Chenouri and Variyath [17], Chenouri et al. [18], Variyath and Vattathoor [19], Variyath and Vattathoor [20] Jobe and Pokojovy [5] and Fan et al. [4].

This article proposed a control chart method that is based on regression adjustment and clustering algorithm for retrospective monitoring of individual characteristics. Since the proposed control chart method blends the addition-point regression with an agglomerative hierarchical cluster algorithm, we refer to it as cluster-regression control chart, (*crcc* for short). Two algorithms are presented for the proposed *crcc* methodology, one to compute the mean vector and covariance matrix in Eqs (2) and (3) and the second one for computing the cluster-regression control chart statistic denoted as $T_{crcc,i}^{2}$ statistic. The notion for the first algorithm is to replace Eqs (2) and (3) with a robust estimate of mean vector and covariance matrix that does not exhibit masking and swamping and can detect certain outlier configurations such as sustained shifts in the mean vector. To account for time *i* in the $T_{crcc,i}^{2}$ second phase algorithm, a projector referred to as “*anchor point matrix*” proposed by Johnson and Wichern [21] and Lawrence et al. [22] is used to trace the data trend through an addition-point least squares regression upon which the cluster distance is constructed. Thus the $T_{crcc,i}^{2}$ is simultaneously able to detect outliers as well as shifts in the mean vector while keeping masking and swamping under control at a given time *i*.

The remaining part of this article is organized in the following way: Section 2 describes the *crcc* methodology and provides algorithms for computing the $T_{crcc,i}^{2}$ control chart. Section 3 deals with construction of the controls limits for the proposed $T_{crcc,i}^{2}$ along with performance evaluation through Monte Carlo simulation experiment. A numerical illustration of artificial dataset generated through a Monte Carlo simulation experiment is closely followed by a real life pulp fiber dataset analysis to implement the *crcc* algorithm in section 4 while section 5 concludes the article.

## The crcc Methodology

The cluster-regression control chart is conceived on the notion that the natural trend of multivariate **X**-quality characteristics can be traced through a projector called “*anchor point matrix*” which can be used to construct a cluster of *h*-observations where $h\geq \frac{\left(n+p+1\right)}{2}$.

Let **m**_**x**_ and $\hat{\text{Σ}}$ be the center and deviation from center of an ellipsoid formed from **X** as defined in Eqs (2) and (3) respectively. Furthermore, let Ω ∈ ℝ^(2*p* + 1) × *p*^ be a set containing the {(2*p* + 1) × *p*} ellipsoid formed from **m**_**x**_ and $\hat{\text{Σ}}$, then define Ω as

$$\begin{array}{c} \Omega =(\begin{array}{c} m_{x}\\ m_{x}\pm \sqrt{\lambda_{i}\chi_{\alpha ,p}^{2}}e_{i} \end{array}). \end{array}$$ (5)

A special case of Ω in which two quality characteristics **x**_1_ and **x**_2_ are measured is defined below. Note that since two quality characteristics are involved, *p* = 2 and {(2*p* + 1) × *p*} becomes {5 × 2}-matrix described below.

$$\begin{array}{c} \Omega =(\begin{array}{c} m_{x}^{\prime }\\ m_{x}^{\prime }-\sqrt{\lambda_{1}\chi_{0.975,2}^{2}}\;\;e_{1}^{\prime }\\ m_{x}^{\prime }+\sqrt{\lambda_{1}\chi_{0.975,2}^{2}}\;\;e_{1}^{\prime }\\ m_{x}^{\prime }-\sqrt{\lambda_{2}\chi_{0.975,2}^{2}}\;\;e_{2}^{\prime }\\ m_{x}^{\prime }+\sqrt{\lambda_{2}\chi_{0.975,2}^{2}}\;\;e_{2}^{\prime } \end{array}) \end{array}$$ (6)

where *λ*_*i*_ and **e**_*i*_ are the eigenvalues and eigenvectors of $\hat{\text{Σ}}$ and $\chi_{\alpha ,p}^{2}$ is the cutoff point of the ellipsoid of constant distance (see Johnson and Wichern [21] for details on Ω). If **m**_**x**_ and $\hat{\text{Σ}}$ are robust, an *OLS* fit to Ω after it has been augmented with the *i* ^*th*^ row of **X** for *n* times (*i* = 1, ⋯, *n*), can mimic the trend of the quality characteristics, **X**. This way, observations that belong to the in-control-process tend to cluster together while those that belong to the out-of-control process as well as those that exhibit certain outlier configuration tend to cluster together and away from those that belong to the in-control-process. The *OLS* fit to Ω after it has been augmented with the *i* ^*th*^ row of **X** for *n* times (*i* = 1, ⋯, *n*) is referred to as “addition point *OLS*”. Since the addition-point *OLS* is performed sequentially, one at a time, by adding one observation from **X** to Ω and fitting *OLS* to it, the time component (*i*) of *T*_*crcc*, *i*_ is preserved. Furthermore, the proper working of Ω requires a robust **m**_**x**_ and $\hat{\text{Σ}}$. Hence, we propose to use a forward search algorithm of Billor et al. [9] to first screen the data of likely outlier configurations and then compute from it, the mean vector, **m**_**x**_ and the covariance matrix, $\hat{\text{Σ}}$ prior to estimation of Ω. Armed with this synopsis, the *crcc* algorithms are presented below.

### The BACON Forward Search Algorithm

Billor et al. [9] proposed an algorithm, the *BACON*: block adaptive computationally efficient outlier nominator, for outlier identification as well as estimator for location and dispersion matrix. The *BACON* algorithm has been used extensively in literature (see [10, 23–25]). Their algorithm select the initial subset denoted as *m* = *p* + 1 as the observations with indices corresponding to the smallest euclidean distance obtained from the coordinatewise median ${\tilde{\text{m}}}_{\text{x}}$.

Having selected the *m* initial subset, the algorithm then proceeds to increase the size of *m* by one observation at a time until the initial subset, *m* contains *h* observations. An observation is nominated as a candidate for *m* if its Mahalanobis distance is the smallest among the observations not included in the *m* initial subset. The algorithm iterates this procedure until all *n* observations are screened. When *h* observations are in *m*, a decision criteria for nominating an outlier is defined, the nominated outliers are removed from **X**, and the mean vector and covariance matrix are computed for the remaining observations in **X** using Eqs (2) and (3). Detailed algorithm is given below.

#### Algorithm 1

**Step 1**: Let ${\tilde{\text{m}}}_{\text{x}}$ be the 1 × *p* vector of coordinatewise median of **X**, compute the distance

$$\begin{array}{c} \tilde{d_{i}}\left(x_{i},\;{\tilde{m}}_{x}\right)=\left|\right|x_{i}-{\tilde{m}}_{x}\left|\right|. \end{array}$$ (7)

**Step 2**: Select the initial subset denoted as *m* with size (*p* + 1) to be the observations with indices corresponding to the smallest distances in (Eq 7). Call this initial subset *m*, “*basic subset*”

**Step 3**: Compute the discrepancies

$$\begin{array}{c} d_{i}\left(x_{i},\;m_{b},\;C_{b}\right)=\sqrt{{\left(x_{i}-m_{b}\right)}^{\prime }C_{b}^{-1}\left(x_{i}-m_{b}\right)},\;i=1,\cdots ,n \end{array}$$ (8)

where **m**_*b*_ and **C**_*b*_ are the mean and covariance matrix of the observations in the basic subset. If **C**_*b*_ is not of full rank, increase the initial subset *m* by adding observations whose indices corresponds to the smallest distances in (Eq 7) until it has full rank.

**Step 4**: Define the new basic subset as all observations whose discrepancy in (8) is less than $c_{npr}\chi_{\left(p,\frac{\alpha }{n}\right)}^{2}$, where $\chi_{\left(p,\frac{\alpha }{n}\right)}^{2}$ is the $\left(1-\frac{\alpha }{n}\right)$ percentile of the chi square distribution with *p* degree of freedom, *c*_*npr*_ = *c*_*np*_ + *c*_*hr*_ is a correction factor, $c_{hr}=max\{0,\frac{\left(h-r\right)}{\left(h+r\right)}\};\;\;h=[\frac{\left(n+p+1\right)}{2}]$, *r* is the size of the current basic subset and

$$\begin{array}{c} c_{np}=1+\frac{p+1}{n-p}+\frac{1}{n-h-p}=1+\frac{p+1}{n-p}+\frac{2}{n-1-3p} \end{array}$$ (9)

See Billor et al. [9] for details on the correction factor.

**Step 5**: Iterate steps 3 and 4 until the size of the basic subset no longer changes.

**Step 6**: Nominate the observations excluded from the final basic subset as outliers

**Step 7**: The location and dispersion estimator is computed as the classical mean and covariance of the observations in the final basic subset using Eqs (2) and (3).

### The crcc Main Algorithm

Let **x**_*ij*_, *i* = 1⋯, *n* *j* = 1, ⋯, *p* be the *j* ^*th*^ quality characteristics in the *i* ^*th*^ sample. The algorithm below computes the proposed $T_{crcc,i}^{2}$ for a given multivariate quality characteristics at time *i*.

#### Algorithm 2

**Step 1**: Compute the mean vector **m**_**x**_ and the covariance matrix, $\hat{\text{Σ}}$ using algorithm 1 above

**Step 2**: Determine a dependent variable, *y* from among the quality characteristics **x**_*ij*_, *i* = 1⋯, *n* *j* = 1, ⋯, *p*. Two choices are available to achieve this. One way is to first compute a covariance matrix from **x**_*ij*_, *i* = 1⋯, *n* *j* = 1, ⋯, *p* and obtain from the covariance matrix, the eigenvalues of each quality characteristics in **x**_*ij*_. The variable or a quality characteristic with the least eigenvalue is nominated as the dependent variance while the remaining (*p* − 1)-variables are treated as regressor variables. The second choice of determining the dependent variable is described below:

- Regress **x**_*j*_ on all the other predictors for *j* = 1, ⋯, *p*. This will yield *p* different regression models for instance, suppose there are 3 quality characteristics denoted as **x**_*i*1_,**x**_*i*2_, and **x**_*i*3_ then the *p* = 3 regression models are
  $$\begin{array}{c} x_{i1}=b_{0}+b_{1}x_{i2}+b_{2}x_{i3} \end{array}$$ (10)
  $$\begin{array}{c} x_{i2}=\alpha_{0}+\alpha_{1}x_{i1}+\alpha_{2}x_{i3} \end{array}$$ (11)
  $$\begin{array}{c} x_{i3}=\beta_{0}+\beta_{1}x_{i1}+\beta_{2}x_{i2} \end{array}$$ (12)
- For all models in Eqs (10), (11) and (12), obtain the corresponding *R* ^2^-values and denote the dependent variable corresponding to the model with the highest *R* ^2^ as the overall dependent variable while the remaining (*p* − 1)-variables are treated as regressors for subsequent addition point *OLS*.

**Step 3**: Construct the {(2*p* + 1) × *p*} projector (anchor-point matrix) as described in Eq (6) above.

**Step 4**: Determine the (*n* × *p*) data matrix **B** with the *i* ^*th*^ row of **B** denoted by a (1 × *p*) vector of **b**_*i*_ to be the estimator that result from an *OLS* regression of Ω, augmented by the *i* ^*th*^ row of **x**_*ij*_.

**Step 5**: Compute an (*n* × *n*) similarity matrix **S** whose elements are defined by

sij=(bi-bj)′(Σ^(B))-1(bi-bj) (13)

The elements of **S** serves as a distance metric upon which an agglomerative hierarchical cluster (*AHC*) analysis of a *complete linkage* is performed on the data. The *AHC* then partition the dataset **x**_*ij*_ into the main cluster *C*_*m*_ containing at least *h* observations and the remaining observations fall into one of *τ* minor clusters labeled as *C*_*τ*1_, *C*_*τ*2_, *C*_*τ*3_, ⋯. See [26–28] for details on cluster analysis.

**Step 6**: Fit a regression model to the observations in the main cluster *C*_*m*_ and obtain from it, the fitted values ${\hat{y}}_{i},i=1,\cdots ,n$ as well as the prediction variance

$$\begin{array}{c} \sigma_{\hat{y}}^{2}=1.4826\underset{\forall i}{m}ed\left|r_{i}-\underset{\forall i}{m}ed\left(r_{i}\right)\right|. \end{array}$$ (14)

where *r*_*i*_ is the residuals from regression model and 1.4826 is a turning constant (see Maronna et al. [29] for details). At this stage, the data points in the minor cluster have not been used and they are said to be inactive. The activation process of the minor cluster is done sequentially, one cluster at a time in the following way:

- Augment the main cluster, *C*_*m*_ with the first minor cluster *C*_*τ*1_ and obtain an *OLS* fitted values denoted as ${\hat{y}}_{i+C_{\tau 1}},i=1,\cdots ,n$
- Obtain the difference in fits statistic, *DFFITS* as
  $$\begin{array}{c} DFFITS_{C_{\tau 1}}=\frac{\sum_{i=1}^{n}{\left({\hat{y}}_{i+C_{\tau 1}}-{\hat{y}}_{i}\right)}^{2}}{n\sigma_{\hat{y}}^{2}}\sim \chi_{\alpha ,p}^{2} \end{array}$$ (15)
- If $DFFITS_{C_{\tau 1}}\leq \chi_{\alpha ,p}^{2}$, then *C*_*τ*1_ minor cluster is included in the main cluster, else, the minor cluster is excluded from **x**_*ij*_ and remain inactive throughout the *crcc* estimation process. This procedure is repeated for all minor clusters. Thus, observation that does not harm the fit produces small value of *DFFITS*_*C*_*τ*1__ and hence, they are activated. However, outliers and mean shift data points tend to produce large values of *DFFITS*_*C*_*τ*1__ and as a result, they are not activated.

**Step 7**: After the minor clusters have been activated by augmenting the main cluster with the minor clusters that satisfy the augmentation condition or otherwise, the mean vector and covariance matrix for *crcc* are then estimated from data points arising from this activation process as

$$\begin{array}{c} m_{x(crcc)}=\frac{1}{n_{a}}\sum_{i=1}^{n_{a}}x_{ij}^{a} \end{array}$$ (16)

and

$$\begin{array}{c} {\hat{\Sigma }}_{\left(crcc\right)}=\frac{1}{n_{a}-1}\sum_{i=1}^{n_{a}}{\left(x_{ij}^{a}-m_{x(crcc)}\right)}^{\prime }\left(x_{ij}^{a}-m_{x(crcc)}\right). \end{array}$$ (17)

where *n*_*a*_ is the sample size of the augmented main cluster and ${\text{x}}_{ij}^{a}$ is the *p*-dimensional multivariate quality characteristics in the current augmented main cluster. The corresponding $T_{crcc,i}^{2}$-control chart plots

$$\begin{array}{c} T_{crcc,i}^{2}={\left(x_{ij}-m_{x(crcc)}\right)}^{\prime }{\hat{\Sigma }}_{\left(crcc\right)}^{-1}\left(x_{ij}-m_{x(crcc)}\right) \end{array}$$ (18)

## Construction of Control Limits and Performance Evaluation of $T_{crcc,i}^{2}$

This section discusses the construction of the control limits of the proposed $T_{crcc,i}^{2}$ as well as examining its performance based on masking and swamping rates. Two other robust control chart methods extensively discussed in literature will be compared with the proposed *T*_*crcc*, *i*_. They are: the re-weighted *MVE* and *MCD* control charts denoted as $T_{RMVE,i}^{2}$ and $T_{RMCD,i}^{2}$ respectively. These methods have been discussed extensively in literature. A few list includes Chenouri and Variyath [17], Chenouri et al. [18], Variyath and Vattathoor [19], Variyath and Vattathoor [20] and Jensen et al. [16].

### Construction of Control Limits Using Simulation Algorithm

In order to implement the proposed $T_{crcc,i}^{2}$ control chart and to also compare the detection performance of various control charts, control limits have to be established first especially when the methods to be considered are based on the robust estimate of mean and variance whose distribution is rarely known. Several approaches have been used in literature for scenarios where the distributions of the control chart parameters are unknown. For instance, Chenouri et al. [18] proposed a robust control chart method in which the classical mean and covariance estimator was replaced with the mean and covariance estimator derived from the re-weighted version of the *MCD* algorithm. They constructed the control limits by using the result of a Monte Carlo simulation experiment to model the empirical distribution of the re-weighted *MCD* algorithm through a family of regression curves. The control limit of their control chart is then estimated from the empirical model at a known value of the dimension *p* and the sample size, *n*. Similar approach of constructing empirical distribution in form of a regression model can also be found in the work of Chenouri and Variyath [17] and Variyath and Vattathoor [19]. This approach works well in detecting little shifts in the mean especially in scenarios where data are contaminated with outliers. A method that is not based on modelling the empirical distribution of the location and dispersion estimators can be found in Jensen et al. [16] and Variyath and Vattathoor [20]. This method constructs the control limits of the robust control charts by iteratively computing the $T_{\left(n\right)}^{2}$-statistic for *i* iterations and then taking the (1 − *α*) 100^*th*^ percentile of the $T_{\left(n\right)}^{2}$-statistic as the established control limits. Our method follow this prescription, with our objective being that the $T_{crcc,i}^{2}$ is able to detect little shift in mean in the presence of outliers. Consequently, the estimation of the control limit for $T_{crcc,i}^{2}$ along with that of $T_{RMVE,i}^{2}$ and $T_{RMCD,i}^{2}$ is described below.

Given an overall false alarm rate of *α*, a control limit (*c*_*α*_) can be obtained from Eq (19)

$$\begin{array}{c} 1-\alpha =p(\underset{1\leq i\leq n}{max}T_{i}^{2}\leq c_{\alpha }|\;\mu =0). \end{array}$$ (19)

Because of the invariance of the $T_{RMVE,i}^{2}$, $T_{RMCD,i}^{2}$ and the $T_{crcc,i}^{2}$ statistics, ***μ*** and **Σ** of the in-control multivariate normal distribution are set to zero vector **0** and identity matrix **I** respectively. Applying Eq (19), the simulation runs for constructing control limits can be performed using the following algorithm:

1. Generate a dataset containing *n* independent observations from *N*_*p*_(**0**,**I**).
2. Compute $T_{i}^{2},i=1,\cdots ,n$ and obtain the largest value $T_{\left(n\right)}^{2}$.
3. Repeat steps 1 and 2 for 100,000 times.
4. Take the (1 − *α*) 100^*th*^ percentile of $T_{\left(n\right)}^{2}$ as the established control limit.

The resulting control limits at various levels of *p* and *n* are presented in Tables 1, 2 and 3 for $T_{crcc,i}^{2}$, $T_{RMVE,i}^{2}$ and $T_{RMCD,i}^{2}$ respectively.

**Table 1:** The simulated control limits for $T_{crcc,i}^{2}$ statistic under various combinations of *n* and *p* at an overall fixed false alarm rate of 0.05.

	*p*								
*n*	2	3	4	5	6	7	8	9	10
10	21.5	28.5	32.6	35.9	39.7	46.8	48.6	64.0	74.1
12	19.1	25.2	26.4	33.0	38.7	43.0	48.8	61.8	72.0
15	18.3	24.2	25.7	31.8	37.1	41.9	46.8	58.0	67.4
20	19.3	21.0	26.1	28.9	36.0	41.8	44.1	57.3	64.9
30	17.1	19.8	26.3	27.9	32.1	36.4	42.0	51.6	57.3
50	16.8	18.5	21.2	23.7	26.8	29.4	33.9	36.1	37.2
62	18.2	20.8	22.0	23.8	26.1	27.6	28.3	31.8	33.1
100	14.1	17.0	19.8	22.5	25.0	26.6	27.9	29.6	31.1

**Table 2:** The simulated control limits for $T_{RMVE,i}^{2}$ statistic under various combinations of *n* and *p* at an overall fixed false alarm rate of 0.05.

	*p*								
*n*	2	3	4	5	6	7	8	9	10
10	24.4	29.0	33.1	36.0	41.6	47.7	49.4	66.2	74.8
12	19.3	24.3	28.8	36.1	39.4	41.8	49.0	63.3	77.2
15	18.7	25.0	26.2	33.0	36.8	42.5	47.9	59.6	71.6
20	19.8	22.4	27.5	28.7	37.4	43.4	46.3	59.2	66.7
30	18.4	21.1	25.8	28.7	33.0	37.0	45.9	53.1	62.5
50	21.2	25.0	29.3	31.5	34.8	38.4	43.6	46.7	51.4
62	21.0	24.3	27.6	29.4	33.0	36.9	41.8	44.8	49.1
100	20.7	24.1	26.5	29.2	32.7	34.4	38.5	40.2	46.0

**Table 3:** The simulated control limits for $T_{RMCD,i}^{2}$ statistic under various combinations of *n* and *p* at an overall fixed false alarm rate of 0.05.

	*p*								
*n*	2	3	4	5	6	7	8	9	10
10	28.2	31.6	35.4	38.5	43.8	49.1	53.4	68.0	77.4
12	20.8	24.0	28.2	38.0	42.3	47.5	52.8	66.7	74.3
15	20.4	23.8	27.7	35.6	38.0	43.6	49.3	61.1	73.4
20	20.1	22.9	27.5	31.0	37.5	43.4	48.8	57.2	64.9
30	19.6	22.6	26.5	29.3	35.1	38.0	47.8	55.0	63.6
50	18.8	26.1	29.0	30.7	34.6	37.3	44.7	48.1	53.5
62	22.6	25.2	28.0	29.9	33.8	37.0	43.1	45.8	53.3
100	21.7	24.6	27.3	29.6	33.0	34.6	40.0	43.8	48.7

As the overall false alarm rate was fixed at 0.05, it can be seen from Tables 1, 2 and 3 that the control limits for the proposed *T*_*crcc*, *i*_ is stable at various levels of *n* and *p* when compared to the $T_{RMVE,i}^{2}$ and $T_{RMCD,i}^{2}$. The $T_{RMVE,i}^{2}$ seems to be moderate when compared to the $T_{RMCD,i}^{2}$ counterpart. It is important to mention here that these limits work well for high dimensions say 5–10. However, with small sample sizes, the control limits may not be reliable.

### Performance Evaluation Through Monte Carlo Simulation Experiment

In order to examine the performance of the proposed *crcc* on effective detection of outliers and mean shift as well as simultaneously minimizing swamping rate, Monte Carlo simulation experiment is performed. In the course of evaluation, the proposed method is compared with other robust multivariate control chart methods such as the *RMVE* and and *RMCD*. The detection rates are used as the yardstick for the performance evaluation. The detection rate is computed as the ratio of the number of correctly identified outliers or mean shift by the methods to the total number of the outliers or mean shift in the dataset. Precisely, let the number of outliers in the dataset be *π* = ⌊*mγ*⌋ and the number of outliers detected by a control chart method at a given non-centrality parameter *λ* ^2^ be *π*(*λ* ^2^), then the detection rate of a method $T_{\ell ,i}^{2},\;\ell =crcc,RMVE,RMCD,i=1,\cdots ,m$ is computed as

$$\begin{array}{c} p\left(T_{\ell ,i}^{2}|\pi \right)=\frac{\pi (\lambda^{2})}{\pi } \end{array}$$ (20)

where *m* is the sample size.

Following the methodology of Variyath and Vattathoor [19], the datasets are generated from a standard multivariate normal distribution *MVN*(**0**, **I**_*p*_) with *r* = 100,000 sample runs of size *m*. Five regimes are considered such that each regime describes the sample size, *m* and the dimension *p*, for instance; Regime 1: {*m* = 30, *p* = 2}; Regime 2: {*m* = 50, *p* = 3}; Regime 3: {*m* = 100, *p* = 5}; Regime 4: {*m* = 150, *p* = 7}; Regime 5: {*m* = 200, *p* = 10}. Within each regime, we considered four levels of mean shifts in data contaminations, namely, *γ* = 0.00, *γ* = 0.10, *γ* = 0.20 and *γ* = 0.25. Without loss of generality, we further assume that a process with the same covariance matrix has been shifted from ***μ***_0_ = 0 to ***μ***_1_ = (*λ*,0, ⋯,0)′ where a non-centrality parameter, *λ* ^2^ = (***μ***_1_ − ***μ***_0_)′ **Σ** ^−1^(***μ***_1_ − ***μ***_0_) represents the size of a process shift taken to be *λ* ^2^ = 0,5,10,15,20. The clean datasets were simulated for observations with the indices *i* = 1, ⋯, *m* − *h* where *h* = ⌊*mγ*⌋ and *γ* is the fraction of data contamination while the contaminated datasets were simulated for observations with the indices *i* = *m* − *h* + 1, ⋯, *m*. The simulation experiment is replicated for *r* = 100,000 runs and for each dataset ${\text{x}}_{ij}^{r}$, the the detection rate is computed using the three methods. The detection rate is expected to be as close as possible to 1 for a method to be classified as performing well. However, the detection rate should close to zero for the null model where *λ* ^2^ = 0. Using Eq (20), the results of the simulation experiment are presented in Tables 4, 5 and 6 for *crcc*, *RMVE* and *RMCD* control charts respectively.

**Table 4:** Simulation result of Performance Evaluation for *crcc* Control Chart.

		Regime				
*λ* ^2^	Outliers	1	2	3	4	5
0	*γ* = 0.00	0.00	0.001	0.000	0.000	0.000
	*γ* = 0.10	0.002	0.001	0.002	0.001	0.000
	*γ* = 0.20	0.013	0.012	0.009	0.008	0.011
	*γ* = 0.25	0.027	0.033	0.041	0.028	0.057
5	*γ* = 0.00	0.763	0.758	0.771	0.699	0.783
	*γ* = 0.10	0.801	0.781	0.808	0.806	0.804
	*γ* = 0.20	0.831	0.772	0.795	0.841	0.844
	*γ* = 0.25	0.847	0.861	0.866	0.870	0.871
10	*γ* = 0.00	0.801	0.813	0.811	0.807	0.809
	*γ* = 0.10	0.805	0.833	0.802	0.701	0.811
	*γ* = 0.20	0.810	0.821	0.815	0.818	0.821
	*γ* = 0.25	0.817	0.828	0.839	0.829	0.839
15	*γ* = 0.00	0.836	0.841	0.838	0.846	0.843
	*γ* = 0.10	0.834	0.836	0.850	0.845	0.856
	*γ* = 0.20	0.812	0.826	0.863	0.871	0.875
	*γ* = 0.25	0.845	0.851	0.855	0.850	0.853
20	*γ* = 0.00	0.906	0.912	0.911	0.916	0.918
	*γ* = 0.10	0.910	0.924	0.924	0.926	0.923
	*γ* = 0.20	0.926	0.928	0.931	0.929	0.927
	*γ* = 0.25	0.935	0.938	0.926	0.958	0.949

**Table 5:** Simulation result of Performance Evaluation for *RMVE* Control Chart.

		Regime				
*λ* ^2^	Outliers	1	2	3	4	5
0	*γ* = 0.00	0.000	0.000	0.001	0.001	0.000
	*γ* = 0.10	0.002	0.001	0.003	0.004	0.001
	*γ* = 0.20	0.021	0.016	0.011	0.014	0.003
	*γ* = 0.25	0.032	0.030	0.032	0.023	0.041
5	*γ* = 0.00	0.467	0.394	0.477	0.463	0.475
	*γ* = 0.10	0.488	0.452	0.480	0.478	0.492
	*γ* = 0.20	0.442	0.470	0.481	0.488	0.492
	*γ* = 0.25	0.479	0.468	0.454	0.473	0.489
10	*γ* = 0.00	0.478	0.484	0.481	0.512	0.514
	*γ* = 0.10	0.501	0.510	0.513	0.488	0.508
	*γ* = 0.20	0.510	0.512	0.503	0.514	0.521
	*γ* = 0.25	0.511	0.520	0.517	0.524	0.522
15	*γ* = 0.00	0.522	0.524	0.516	0.523	0.526
	*γ* = 0.10	0.530	0.536	0.528	0.529	0.540
	*γ* = 0.20	0.632	0.633	0.642	0.641	0.636
	*γ* = 0.25	0.703	0.710	0.709	0.713	0.711
20	*γ* = 0.00	0.803	0.824	0.824	0.818	0.828
	*γ* = 0.10	0.826	0.831	0.845	0.851	0.857
	*γ* = 0.20	0.892	0.903	0.902	0.904	0.914
	*γ* = 0.25	0.920	0.919	0.901	0.921	0.926

**Table 6:** Simulation result of Performance Evaluation for *RMCD* Control Chart.

		Regime				
*λ* ^2^	Outliers	1	2	3	4	5
0	*γ* = 0.00	0.001	0.001	0.001	0.000	0.001
	*γ* = 0.10	0.002	0.003	0.003	0.001	0.0002
	*γ* = 0.20	0.014	0.022	0.011	0.031	0.021
	*γ* = 0.25	0.022	0.031	0.043	0.042	0.052
5	*γ* = 0.00	0.321	0.318	0.309	0.313	0.320
	*γ* = 0.10	0.320	0.322	0.323	0.325	0.336
	*γ* = 0.20	0.434	0.427	0.431	0.438	0.429
	*γ* = 0.25	0.449	0.438	0.454	0.447	0.449
10	*γ* = 0.00	0.442	0.438	0.441	0.451	0.451
	*γ* = 0.10	0.451	0.435	0.453	0.438	0.500
	*γ* = 0.20	0.471	0.461	0.465	0.466	0.473
	*γ* = 0.25	0.481	0.472	0.473	0.482	0.478
15	*γ* = 0.00	0.502	0.504	0.511	0.514	0.513
	*γ* = 0.10	0.553	0.566	0.567	0.566	0.564
	*γ* = 0.20	0.733	0.716	0.746	0.736	0.751
	*γ* = 0.25	0.730	0.731	0.739	0.743	0.761
20	*γ* = 0.00	0.840	0.840	0.845	0.848	0.851
	*γ* = 0.10	0.847	0.846	0.849	0.860	0.862
	*γ* = 0.20	0.891	0.895	0.911	0.914	0.914
	*γ* = 0.25	0.923	0.921	0.920	0.924	0.928

From Tables 4, 5 and 6, it can be seen that:

1. At the null model ie *λ* ^2^ = 0, all methods considered performed quite well and hence, they did not identify any point as outliers and/or out-of-control.
2. The *crcc* has a higher detection rate compared to the *RMVE* and *RMCD*. This is noticeable when the non-centrality parameter is small (say 5 ≤ *λ* ^2^ ≤ 10). Hence, the *crcc* is sensitive in detecting small shift in the in-control mean when outliers are present in datasets
3. The *RMVE* and *RMCD* performed quite well especially when the non-centrality parameter is large (say *λ* ^2^ > 10)
4. In all scenarios considered, the sample size *m* and the dimension *p* has little or no effect on the detection rate.
5. The detection rate of all methods considered increases as the non-centrality parameter (mean shift = *λ* ^2^) and the level of contamination (outliers = *γ*) increases. Hence, *λ* ^2^ and *γ* are the two parameters that influences the detection rate.

## Numerical Implementation and Real Life Data Application of *crcc* Algorithm

### Artificial Data

In order to facilitate the understanding of *crcc* algorithm and the working mechanisms, a follow up numerical illustration is given below. Following the methodology of Lawrence et al. [22], two variable quality characteristics of sample size 12 is simulated in the following way: Observations 1–9 comes from *x*_*i*2_ = 200 − 4*x*_*i*1_ + *e*_*i*_ and *x*_*i*1_ ∼ *U*[10; 20] with the error term *e*_*i*_ ∼ *N*(*μ* = 0, *σ* = 5). Observations 10–12 are outliers deliberately planted such that: for 10 ≤ *i* ≤ 12, *x*_*i*1_ = (29, 30, 31) and *x*_*i*2_ = (153.800, 155.800, 80.932). The data arising from the process is presented in Table 7 below

**Table 7:** Two Variable Artificial Dataset.

*i*	*x*_*i*1_	*x*_*i*2_
1	9.483	167.611
2	9.772	162.155
3	11.376	154.880
4	12.383	144.862
5	14.754	148.578
6	15.160	144.966
7	14.771	137.416
8	13.585	141.657
9	15.446	134.214
10	29.000	153.800
11	30.000	155.800
12	31.000	80.932

The stepwise implementation of *crcc* algorithm on the two variable artificial data is presented below.

**Step 1**: Given that *p* = 2, *n* = 12, and *h* = ⌊(*n* + *p* + 1)/2⌋ = 7, the (1 × 2) mean vector **m**_**x**_ and the (2 × 2) covariance matrix computed from algorithm 1 in section 2.1.1 are:

$$\begin{array}{c} m_{x}=(\begin{array}{cc} 12.9698 & 148.4821 \end{array}) \end{array}$$ (21)

and

$$\begin{array}{c} \hat{\Sigma }=(\begin{array}{cc} 5.3757 & -23.1865\\ -23.1865 & 123.9866 \end{array}) \end{array}$$ (22)

**Step 2**: The eigenvalues of ***x***_*i*1_ and ***x***_*i*2_ denoted as *λ*_1_ and *λ*_2_, computed from the covariance matrix, $\hat{\text{Σ}}$ in Eq (3) are

$$\begin{array}{c} \lambda =(\begin{array}{cc} \lambda_{1} & \lambda_{2}\\ 128.3580 & 1.0042 \end{array}) \end{array}$$ (23)

since **x**_*i*2_ has the least eigenvalue of *λ*_2_ = 1.0042, it is nominated and denoted as the dependent variable, *y*_*i*_.

**Step 3**: The eigenvectors of **x**_*i*1_ and **x**_*i*2_ denoted as **e**_1_ and **e**_2_ computed from the covariance matrix, $\hat{\text{Σ}}$ in Eq (3) are

$$\begin{array}{c} E=(\begin{array}{cc} e_{1} & e_{2}\\ -0.1853 & -0.9827\\ 0.9827 & -0.1853 \end{array}) \end{array}$$ (24)

with $\chi_{0.975,2}^{2}=7.3777$ and hence the (5 × 2) projector matrix described in Eq (6) is given as

$$\begin{array}{c} \Omega =[\begin{array}{c} m_{x}^{\prime }\\ m_{x}^{\prime }-\sqrt{\lambda_{1}\chi_{0.975,2}^{2}}\;\;e_{1}^{\prime }\\ m_{x}^{\prime }+\sqrt{\lambda_{1}\chi_{0.975,2}^{2}}\;\;e_{1}^{\prime }\\ m_{x}^{\prime }-\sqrt{\lambda_{2}\chi_{0.975,2}^{2}}\;\;e_{2}^{\prime }\\ m_{x}^{\prime }+\sqrt{\lambda_{2}\chi_{0.975,2}^{2}}\;\;e_{2}^{\prime } \end{array}]=\left[\begin{array}{cc} 12.9698 & 148.4821\\ 18.6712 & 118.2416\\ 7.2684 & 178.7226\\ 15.6445 & 148.9864\\ 10.2950 & 147.9778 \end{array}\right] \end{array}$$ (25)

**Step 4**: The (12 × 2) data matrix *B* resulting from an *OLS* regression of Ω augmented by the *i* ^*th*^ row of **x**_*ij*_ is computed this way: Add the first row of the data in Table 7 to Ω to obtain the data in Table 8.

**Table 8:** Augmented Projector.

*i*	*x*_*i*1_	*x*_*i*2_
1	12.9698	148.4821
2	18.6712	118.2416
3	7.2684	178.7226
4	15.6445	148.9864
5	10.2950	147.9778
6	9.483	167.611

Notice that Ω has become 6 × 2 matrix because row 1 of Table 7 has been merged with Ω in Eq (25). An *OLS* fit to the data in Table 8 result in the estimates **b**_1_ = 200.7503, −4.4460. Perform this operation for *n* = 12 times. Note that the augmentation is done without replacement. The resulting estimate is presented in Table 9 below

**Table 9:** Addition-point *OLS* matrix, *B*.

*i*	**b**_*i*0_	**b**_*i*1_
1	206.750	−4.446
2	204.358	−4.310
3	204.246	−4.305
4	202.912	−4.275
5	203.848	−4.172
6	203.680	−4.183
7	204.675	−4.374
8	204.080	−4.340
9	204.999	−4.401
10	163.818	−0.924
11	161.436	−0.742
12	199.124	−3.875

**Step 5**: The (*n* × *n*) similarity matrix computed for the data in Table 9, using Eq (13) is presented in Table 10 below

**Table 10:** Similarity Matrix $s_{ij}={\left({\text{b}}_{i}-{\text{b}}_{j}\right)}^{\prime }{\left(\hat{\text{Σ}}(\text{B})\right)}^{-1}({\text{b}}_{i}-{\text{b}}_{j})$.

**S**_*ij*_	**1**	**2**	**3**	**4**	**5**	**6**	**7**	**8**	**9**	**10**	**11**	**12**
**1**	0	5.146	5.671	14.842	9.314	9.291	4.948	7.644	4.086	1,750	1,944	52
**2**	5.146	0	0.014	2.893	3.280	2.281	0.613	0.743	1.040	1,584	1,768	26
**3**	5.671	0.014	0	2.508	3.388	2.314	0.609	0.611	1.089	1,577	1,761	25
**4**	14.842	2.893	2.508	0	8.476	6.274	2.818	1.239	3.863	1,508	1,687	18
**5**	9.314	3.280	3.388	8.476	0	0.167	6.709	6.644	7.775	1,509	1,690	19
**6**	9.291	2.281	2.314	6.274	0.167	0	5.258	4.944	6.344	1,505	1,685	18
**7**	4.948	0.613	0.609	2.818	6.709	5.258	0	0.320	0.102	1,624	1,810	31
**8**	7.644	0.743	0.611	1.239	6.644	4.944	0.320	0	0.741	1,584	1,768	26
**9**	4.086	1.040	1.089	3.863	7.775	6.344	0.102	0.741	0	1,650	1,838	35
**10**	1,750	1,584	1,577	1,508	1,509	1,505	1,624	1,584	1,650	0	5.143	1,202
**11**	1,944	1,768	1,761	1,687	1,690	1,685	1,810	1,768	1,838	5	0	1,363
**12**	52	26	25.432	18	19	18	31	26	35	1,202	1,363	0

**Fig 1:**
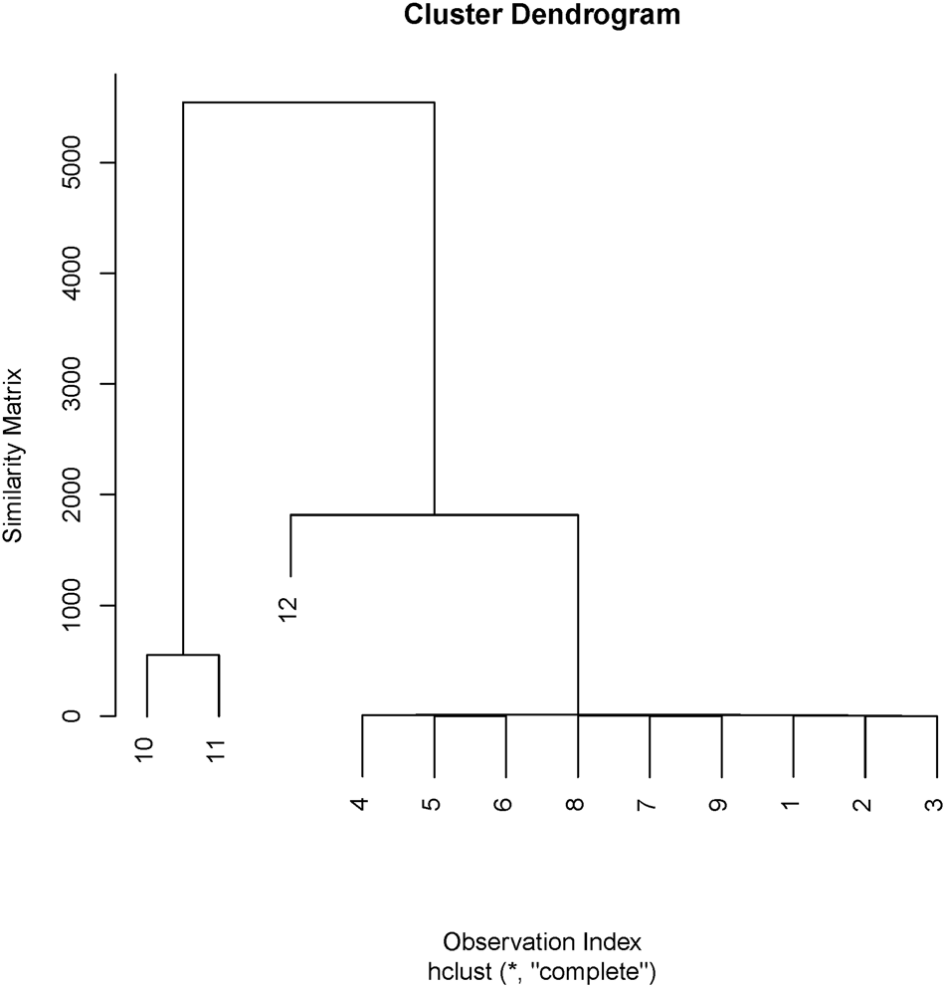
Dendrogram Plot of Artificial Data.

The agglomerative hierarchical cluster analysis based on the similarity matrix produces the dendrogram plot in Fig 1 below.

Notice that the 3 outliers cluster differently while the inliers cluster together. The plot shows that the main cluster *C*_*M*_ contains observations 1:9 while the 3 minor clusters are *C*_*τ*1_ = {10}, *C*_*τ*2_ = {11}, *C*_*τ*3_ = {12}

**Step 6**: An *OLS* fit the the data points in the main cluster yields the estimates **b**_*j*_ = 204.4238, −4.3132 and the corresponding prediction variance computed using Eq (14) is $\sigma_{\hat{y}}^{2}=8.4338$. The 3 minor clusters are investigated for likely activation through the *DFFITS*_*τi*_. Their corresponding *DFFITS*_*τi*_ as well as the cutoff value are

$$\begin{array}{c} \left(\begin{array}{cccc} Minor\;Clusters= & C_{\tau 1} & C_{\tau 2} & C_{\tau 3}\\ DFFITS= & 603.9871 & 647.8526 & 7.1054^{*}\\ \chi_{0.975,2}^{2}= & 7.3778 & 7.3778 & 7.3778 \end{array}\right) \end{array}$$ (26)

Notice that the DFIITS-statistics for the third cluster, *C*_*τ*3_ is less than the cutoff value. Hence cluster 3 with observation number 12 is activated while cluster 1 and 2 with observations numbers 10 and 11 respectively, remain inactive.

**Step 7**: Having activated cluster 3, *n*_*a*_ = 10, the *crcc* control chart parameters are computed thus:

$$\begin{array}{c} m_{x(crcc)}=(\begin{array}{cc} 12.7468 & 148.4701 \end{array}) \end{array}$$ (27)

and

$$\begin{array}{c} {\hat{\Sigma }}_{\left(crcc\right)}=(\begin{array}{cc} 5.6323 & -26.5263\\ -26.5263 & 141.6975 \end{array}). \end{array}$$ (28)

The resulting control chart statistic, $T_{crcc,i}^{2}$ alongside two robust control chart methods such as the *RMVE* control chart, $T_{RMVE,i}^{2}$, the *RMCD* control chart, $T_{RMCD,i}^{2}$ and the classical Hotelling $T_{i}^{2}$ control chart statistics are presented in Table 11 below.

**Table 11:** The *crcc*, *RMVE*, *RMCD*, and classical Hotelling’s *T* ^2^ statistics.

$T_{crcc,i}^{2}$	$T_{RMVE,i}^{2}$	$T_{RMCD,i}^{2}$	$T_{i}^{2}$
2.738	18.756	17.658	1.407
1.578	3.622	5.954	1.049
0.334	0.232	1.236	0.563
1.714	2.699	2.890	0.458
6.165	19.750	21.395	0.101
4.721	5.568	6.600	0.076
0.865	1.118	2.281	0.359
0.614	0.634	1.585	0.352
1.436	2.814	3.493	0.449
446.753	77,857.170	64,857.820	4.028
520.922	106,309.400	112,846.600	4.922
79.413	4,202.813	5,017.478	8.236

**Fig 2:**
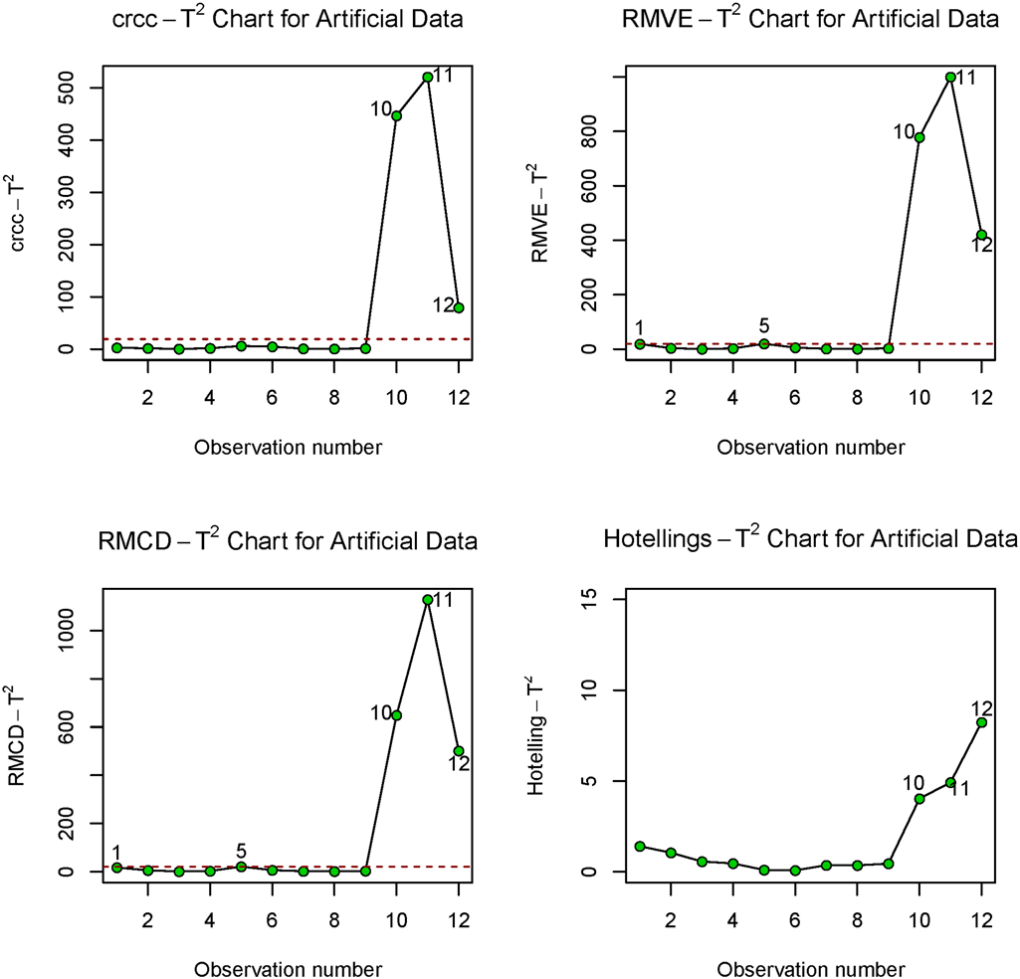
Control Charts for Artificial Data.

The corresponding control chart for the four methods is presented in Fig 2 below

Notice from Table 11 and Fig 2 above that the *crcc* control chart is able to detect the 3 spurious outliers planted at index 10, 11 and 12 as an out-of-control points. The *crcc* control chart has the feature to detect the in-control-trend while the dendrogram plot in Fig 1 also depict the outlier structure in multivariate data. The *RMVE* and *RMDC* were able to detect observations 10–12 as an out-of-control point. However, due to swamping effect, they erroneously nominated observations 1 and 5 which were originally in-control-points as out-of-control points. Surprisingly, the classical Hotelling *T* ^2^ computed from the function *qcc* in *R* language could not even identify any of the 3 mean shift outlier points. This is because observations 10–12 attracted the mean to themselves and away from the other in-control observations so that the covariance matrix becomes arbitrarily large and the squared Mahalanobis distances computed based on these mean and covariance becomes arbitrarily small and hence, the 3 observations are masked.

### The Pulp Fiber Real-life Dataset

Following the experimental design of Lee [30], Rousseeuw et al. [31] describe an experiment that was conducted to determine the effect of pulp fiber properties on the paper made from them. The pulp fiber properties that were measured for paper production are: mean fiber length, *x*_*i*1_, long fiber fraction, *x*_*i*2_, fine fiber fraction, *x*_*i*3_, and zero span tensile, *x*_*i*4_. The paper properties that were measured after production are: breaking length, *y*_*i*1_, elastic modulus, *y*_*i*2_, stress at failure, *y*_*i*3_, and burst strength, *y*_*i*4_. The dataset arising from this process contains *n* = 62 measurements as presented in Table 12.

**Table 12:** The Pulp-fibre Dataset.

*i*	*x*_1*i*_	*x*_2*i*_	*x*_3*i*_	*x*_4*i*_	*y*_1*i*_	*y*_2*i*_	*y*_3*i*_	*y*_4*i*_
1	-0.030	35.239	36.991	1.057	21.312	7.039	5.326	0.932
2	0.015	35.713	36.851	1.064	21.206	6.979	5.237	0.871
3	0.025	39.220	30.586	1.053	20.709	6.779	5.060	0.742
4	0.030	39.756	21.072	1.050	19.542	6.601	4.479	0.513
5	-0.070	32.991	36.570	1.049	20.449	6.795	4.912	0.577
6	-0.050	31.140	38.115	1.052	20.841	6.919	5.108	0.784
7	-0.247	28.375	41.364	1.044	19.060	6.447	4.246	0.358
8	-0.099	32.580	36.430	1.038	18.597	6.261	4.032	0.215
9	-0.242	23.889	49.080	1.042	19.346	6.572	4.358	0.432
10	-0.188	28.027	39.243	1.042	18.720	6.455	4.072	0.372
11	-0.099	33.128	32.802	1.052	18.587	6.295	4.068	0.239
12	-0.232	26.492	40.939	1.042	19.813	6.775	4.604	0.637
13	-0.045	32.169	32.524	1.045	19.989	6.737	4.686	0.779
14	0.055	35.103	31.139	1.042	19.116	6.512	4.299	0.588
15	0.070	40.893	21.473	1.049	18.769	6.335	4.089	0.470
16	-0.015	32.649	31.554	1.038	18.708	6.271	3.978	0.457
17	-0.109	27.749	38.538	1.036	19.323	6.550	4.404	0.588
18	0.000	36.187	25.927	1.022	17.433	5.948	3.486	0.104
19	-0.193	34.491	25.519	1.047	19.195	6.213	4.300	0.405
20	-0.090	31.827	29.209	1.050	19.436	6.387	4.404	0.519
21	-0.154	29.622	32.385	1.057	20.136	6.725	4.723	0.652
22	-0.154	35.917	29.346	1.033	16.740	6.168	3.201	0.104
23	-0.149	30.658	35.730	1.033	18.589	6.531	3.989	0.336
24	-0.271	29.415	33.775	1.033	19.422	6.615	4.382	0.432
25	0.243	51.638	15.922	1.099	24.420	7.874	6.999	1.730
26	0.340	58.686	9.159	1.101	25.288	8.034	7.406	1.873
27	0.080	49.025	27.700	1.097	26.119	8.222	7.771	1.946
28	0.131	46.266	23.893	1.076	23.113	7.288	6.329	1.513
29	0.136	50.333	17.888	1.095	25.209	7.955	7.296	1.792
30	0.176	44.218	26.880	1.090	25.444	8.045	7.477	1.847
31	0.151	43.887	33.775	1.082	23.699	7.593	6.609	1.482
32	0.207	48.894	23.219	1.081	24.303	7.775	6.861	1.583
33	-0.015	40.158	42.074	1.066	24.793	8.123	7.202	1.703
34	0.126	54.559	11.293	1.089	23.438	7.650	6.457	1.477
35	0.131	49.025	17.494	1.088	24.197	7.794	6.833	1.583
36	0.070	49.287	23.354	1.092	24.741	7.996	7.152	1.728
37	0.156	45.673	29.622	1.070	24.170	7.766	6.846	1.615
38	0.055	45.475	21.000	1.076	24.174	7.877	6.826	1.692
39	-0.015	42.958	33.636	1.085	25.052	8.287	7.332	1.773
40	0.090	48.632	13.977	1.070	23.846	7.639	6.615	1.560
41	0.015	49.025	18.284	1.073	24.822	8.041	7.129	1.721
42	0.010	43.821	27.290	1.087	25.200	7.356	7.356	1.785
43	0.131	46.530	18.284	1.069	23.695	7.460	6.567	1.543
44	0.000	46.398	18.416	1.075	24.941	7.929	7.286	1.703
45	-0.099	44.946	24.164	1.078	25.007	8.081	7.287	1.787
46	-0.188	51.898	19.209	1.064	21.183	7.156	5.388	0.924
47	-0.173	48.436	26.880	1.065	21.875	7.336	5.762	1.068
48	-0.227	47.254	29.346	1.066	22.095	7.447	5.790	1.182
49	0.314	56.627	2.925	1.118	25.166	7.913	7.211	1.813
50	0.217	53.458	0.511	1.122	24.560	7.854	7.020	1.701
51	0.381	60.993	0.000	1.118	22.007	8.259	7.322	1.169
52	0.397	58.429	1.147	1.129	21.115	7.913	6.557	0.928
53	0.289	56.755	0.407	1.113	26.194	8.454	7.816	2.145
54	0.202	56.111	0.407	1.104	25.674	8.208	7.534	2.046
55	0.273	53.847	2.023	1.111	25.930	8.100	7.669	2.037
56	0.558	63.035	-0.391	1.113	21.390	7.475	5.294	0.875
57	-0.672	3.448	76.878	1.020	18.441	6.652	3.946	0.140
58	-0.605	2.845	84.554	1.008	16.441	6.315	2.997	-0.400
59	-0.694	1.515	81.988	0.998	16.294	6.572	3.017	-0.478
60	-0.559	2.054	8.786	1.081	20.289	7.719	4.866	0.239
61	-0.415	3.018	5.855	1.033	17.163	7.086	3.396	-0.236
62	-0.324	17.639	28.934	1.070	20.289	7.437	4.859	0.470

The *crcc* step-wise algorithm for the pulp-fiber data is presented below.

**Step 1**: Given that *p* = 8, and *h* = ⌊(*n* + *p* + 1)/2⌋ = 35, the (1 × 8) mean vector **m**_**x**_ and the (8 × 8) covariance matrix computed from algorithm 1 in section 2.1.1 are:

$$\begin{array}{c} m_{x}=(\begin{array}{cccccccc} 0.0188 & 40.7620 & 26.8214 & 1.0661 & 22.1054 & 7.2565 & 5.7775 & 1.1373 \end{array}) \end{array}$$ (29)

and

$$\begin{array}{c} \hat{\Sigma }=(\begin{array}{cccccccc} 0.0240 & 1.3352 & -1.4181 & 0.0031 & 0.3308 & 0.0802 & 0.1669 & 0.0785\\ 1.3352 & 91.8882 & -96.6598 & 0.2094 & 23.3711 & 5.9196 & 11.8213 & 5.4771\\ -1.4181 & -96.6598 & 131.9636 & -0.2097 & -20.5775 & -5.1145 & -10.3236 & -5.0097\\ 0.0031 & 0.2094 & -0.2097 & 0.0006 & 0.0636 & 0.0161 & 0.0321 & 0.0146\\ 0.3308 & 23.3711 & -20.5775 & 0.0636 & 8.0646 & 2.0611 & 4.0674 & 1.8301\\ 0.0802 & 5.9196 & -5.1145 & 0.0161 & 2.0611 & 0.5391 & 1.0415 & 0.4690\\ 0.1669 & 11.8213 & -10.3236 & 0.0321 & 4.0674 & 1.0415 & 2.0532 & 0.9237\\ 0.0785 & 5.4771 & -5.0097 & 0.0146 & 1.8301 & 0.4690 & 0.9237 & 0.4216 \end{array}) \end{array}$$ (30)

**Step 2**: The eigenvalues of ***x***_*i*1_, ***x***_*i*2_, ***x***_*i*3_, ***x***_*i*4_, ***x***_*i*5_, ***x***_*i*6_, ***x***_*i*7_ and ***x***_*i*8_ denoted as *λ*_1_, *λ*_2_, *λ*_3_, *λ*_4_, *λ*_5_, *λ*_6_, *λ*_7_ and *λ*_8_, computed from the covariance matrix, $\hat{\text{Σ}}$ in Eq (30) are

$$\begin{array}{c} \text{λ}=(\begin{array}{cccccccc} \lambda_{1} & \lambda_{2} & \lambda_{3} & \lambda_{4} & \lambda_{5} & \lambda_{6} & \lambda_{7} & \lambda_{8}\\ 216.9587 & 16.2786 & 1.6964 & 0.0128 & 0.0053 & 0.0024 & 7e^{-4} & 3.8e^{-5} \end{array}) \end{array}$$ (31)

since **x**_*i*8_ has the least eigenvalue of *λ*_8_ = 3.8*e* ^−5^, it is nominated and denoted as the dependent variable, *y*_*i*_ and the remaining variables are treated as regressors at the regression stage of *crcc*.

**Step 3**: The eigenvectors of **x**_*i*1_, **x**_*i*2_, **x**_*i*3_, **x**_*i*4_, **x**_*i*5_, **x**_*i*6_, **x**_*i*7_ and **x**_*i*8_ denoted as **e**_1_, **e**_2_, **e**_3_, **e**_4_, **e**_5_, **e**_6_, **e**_7_ and **e**_8_ computed from the covariance matrix, $\hat{\text{Σ}}$ in Eq (30) are

$$\begin{array}{c} E=(\begin{array}{cccccccc} e_{1} & e_{2} & e_{3} & e_{4} & e_{5} & e_{6} & e_{7} & e_{8}\\ -0.0094 & -0.0080 & 0.0244 & 0.2296 & 0.7439 & 0.5931 & -0.1990 & -0.0427\\ -0.5936 & -0.6629 & 0.4557 & -0.0098 & -0.0146 & -0.0119 & 0.0090 & 0.0005\\ 0.7944 & -0.5731 & 0.2011 & 0.0036 & 0.0012 & -0.0050 & 0.0039 & 0.0006\\ -0.0011 & -0.0026 & -0.0046 & 0.0035 & 0.0082 & 0.0712 & 0.0326 & 0.9969\\ -0.1099 & -0.4085 & -0.7380 & 0.2782 & -0.0966 & 0.1687 & 0.4004 & -0.0299\\ -0.0248 & -0.1166 & -0.2128 & -0.9291 & 0.0988 & 0.2555 & 0.0424 & -0.0185\\ -0.0550 & -0.2096 & -0.3662 & 0.0194 & -0.1205 & -0.1393 & -0.8850 & 0.0375\\ -0.0272 & -0.0874 & -0.1651 & -0.0778 & 0.6425 & -0.7279 & 0.1179 & 0.0421 \end{array}) \end{array}$$ (32)

with $\chi_{0.975,8}^{2}=17.5346$ and hence the (17 × 8) projector matrix described in Eq (6) is given as

$$\begin{array}{c} \Omega =[\begin{array}{c} m_{x}^{\prime }\\ m_{x}^{\prime }-\sqrt{\lambda_{1}\chi_{0.975,8}^{2}}\;\;e_{1}^{\prime }\\ m_{x}^{\prime }+\sqrt{\lambda_{1}\chi_{0.975,8}^{2}}\;\;e_{1}^{\prime }\\ m_{x}^{\prime }-\sqrt{\lambda_{2}\chi_{0.975,8}^{2}}\;\;e_{2}^{\prime }\\ m_{x}^{\prime }+\sqrt{\lambda_{2}\chi_{0.975,8}^{2}}\;\;e_{2}^{\prime }\\ m_{x}^{\prime }+\sqrt{\lambda_{3}\chi_{0.975,8}^{2}}\;\;e_{3}^{\prime }\\ m_{x}^{\prime }+\sqrt{\lambda_{3}\chi_{0.975,8}^{2}}\;\;e_{3}^{\prime }\\ m_{x}^{\prime }+\sqrt{\lambda_{4}\chi_{0.975,8}^{2}}\;\;e_{4}^{\prime }\\ m_{x}^{\prime }+\sqrt{\lambda_{4}\chi_{0.975,8}^{2}}\;\;e_{4}^{\prime }\\ m_{x}^{\prime }+\sqrt{\lambda_{5}\chi_{0.975,8}^{2}}\;\;e_{5}^{\prime }\\ m_{x}^{\prime }+\sqrt{\lambda_{5}\chi_{0.975,8}^{2}}\;\;e_{5}^{\prime }\\ m_{x}^{\prime }+\sqrt{\lambda_{6}\chi_{0.975,8}^{2}}\;\;e_{6}^{\prime }\\ m_{x}^{\prime }+\sqrt{\lambda_{6}\chi_{0.975,8}^{2}}\;\;e_{6}^{\prime }\\ m_{x}^{\prime }+\sqrt{\lambda_{7}\chi_{0.975,8}^{2}}\;\;e_{7}^{\prime }\\ m_{x}^{\prime }+\sqrt{\lambda_{7}\chi_{0.975,8}^{2}}\;\;e_{7}^{\prime }\\ m_{x}^{\prime }+\sqrt{\lambda_{8}\chi_{0.975,8}^{2}}\;\;e_{8}^{\prime }\\ m_{x}^{\prime }+\sqrt{\lambda_{8}\chi_{0.975,8}^{2}}\;\;e_{8}^{\prime } \end{array}]=\left[\begin{array}{cccccccc} -0.0229 & 38.3767 & 30.2106 & 1.0613 & 21.7245 & 7.1597 & 5.5869 & 1.0313\\ 0.7067 & 85.7393 & -30.4340 & 1.1419 & 30.1144 & 9.0034 & 9.8044 & 3.1229\\ -0.7525 & -8.9858 & 90.8553 & 0.9808 & 13.3345 & 5.3159 & 1.3694 & -1.0603\\ 0.1197 & 49.6032 & 40.3845 & 1.1099 & 29.1127 & 9.2708 & 9.3733 & 2.6172\\ -0.1655 & 27.1503 & 20.0368 & 1.0127 & 14.3362 & 5.0486 & 1.8006 & -0.5546\\ -0.1853 & 36.0139 & 29.0697 & 1.0795 & 25.4585 & 8.2187 & 7.4383 & 1.8651\\ 0.1395 & 40.7396 & 31.3516 & 1.0432 & 17.9904 & 6.1006 & 3.7356 & 0.1974\\ 0.1072 & 38.3719 & 30.2125 & 1.0637 & 21.8314 & 6.7609 & 5.5962 & 1.0524\\ -0.1530 & 38.3816 & 30.2088 & 1.0590 & 21.6175 & 7.5585 & 5.5777 & 1.0102\\ 0.1679 & 38.3724 & 30.2106 & 1.0611 & 21.6756 & 7.2207 & 5.5547 & 1.2690\\ -0.2137 & 38.3811 & 30.2107 & 1.0616 & 21.7733 & 7.0987 & 5.6192 & 0.7936\\ -0.1609 & 38.3800 & 30.2119 & 1.0554 & 21.6910 & 7.1128 & 5.6199 & 1.1517\\ 0.1151 & 38.3734 & 30.2093 & 1.0673 & 21.7579 & 7.2066 & 5.5539 & 0.9109\\ 0.0054 & 38.3754 & 30.2100 & 1.0589 & 21.6701 & 7.1562 & 5.7065 & 1.0145\\ -0.0512 & 38.3781 & 30.2112 & 1.0637 & 21.7788 & 7.1631 & 5.4674 & 1.0481\\ -0.0224 & 38.3767 & 30.2106 & 1.0336 & 21.7249 & 7.1598 & 5.5864 & 1.0308\\ -0.0234 & 38.3767 & 30.2106 & 1.0890 & 21.7240 & 7.1596 & 5.5874 & 1.0317 \end{array}\right] \end{array}$$ (33)

**Step 4**: The (62 × 8) data matrix *B* resulting from an *OLS* regression of Ω augmented by the *i* ^*th*^ row of **x**_*ij*_ is presented in Table 13

**Table 13:** Addition-Point *OLS* matrix *B* for Pulp-fibre Dataset.

*i*	*x*_1*i*_	*x*_2*i*_	*x*_3*i*_	*x*_4*i*_	*y*_1*i*_	*y*_2*i*_	*y*_3*i*_	*y*_4*i*_
1	−0.544	0.493	−0.009	−0.006	−2.039	0.077	0.191	0.218
2	−0.388	0.490	−0.009	−0.006	−2.054	0.065	0.190	0.243
3	−0.430	0.497	−0.010	−0.006	−1.988	0.059	0.204	0.250
4	−0.582	0.479	−0.009	−0.006	−1.901	0.075	0.170	0.238
5	−0.196	0.480	−0.009	−0.006	−1.969	0.036	0.198	0.296
6	−0.425	0.463	−0.008	−0.006	−2.012	0.063	0.187	0.246
7	−0.479	0.483	−0.009	−0.006	−2.001	0.069	0.188	0.236
8	−0.582	0.518	−0.010	−0.006	−2.132	0.083	0.214	0.201
9	−0.501	0.489	−0.009	−0.006	−2.004	0.071	0.190	0.231
10	−0.525	0.484	−0.009	−0.006	−1.931	0.064	0.197	0.240
11	−0.553	0.514	−0.009	−0.006	−2.407	0.108	0.204	0.158
12	−0.521	0.486	−0.010	−0.006	−2.007	0.070	0.203	0.228
13	−0.285	0.543	−0.011	−0.006	−2.259	0.072	0.212	0.222
14	−0.178	0.540	−0.010	−0.006	−2.150	0.050	0.209	0.262
15	−0.462	0.492	−0.008	−0.006	−2.047	0.069	0.195	0.228
16	−0.694	0.515	−0.009	−0.006	−2.262	0.115	0.188	0.145
17	−0.075	0.525	−0.011	−0.006	−2.143	0.048	0.194	0.280
18	−0.830	0.484	−0.009	−0.006	−1.867	0.086	0.195	0.200
19	−0.425	0.468	−0.009	−0.006	−2.006	0.070	0.173	0.241
20	−0.430	0.488	−0.009	−0.006	−2.022	0.070	0.183	0.238
21	−0.467	0.490	−0.009	−0.006	−2.029	0.070	0.190	0.233
22	−0.222	0.447	−0.007	−0.005	−1.732	−0.011	0.260	0.341
23	−0.423	0.489	−0.009	−0.006	−2.013	0.068	0.180	0.243
24	−0.415	0.523	−0.009	−0.006	−1.914	0.055	0.189	0.263
25	−0.407	0.502	−0.009	−0.006	−1.948	0.056	0.195	0.258
26	−0.470	0.482	−0.010	−0.006	−2.038	0.073	0.187	0.230
27	−0.447	0.491	−0.009	−0.006	−2.042	0.069	0.190	0.235
28	0.279	0.462	−0.008	−0.005	−1.909	0.029	0.079	0.369
29	−0.425	0.493	−0.009	−0.006	−2.045	0.067	0.192	0.238
30	−0.680	0.447	−0.008	−0.006	−2.013	0.081	0.199	0.200
31	−0.468	0.497	−0.009	−0.006	−1.992	0.067	0.187	0.240
32	−0.363	0.467	−0.010	−0.006	−1.904	0.052	0.187	0.271
33	−0.492	0.487	−0.009	−0.006	−2.011	0.073	0.186	0.230
34	−0.662	0.456	−0.008	−0.005	−1.938	0.072	0.198	0.218
35	−0.424	0.493	−0.009	−0.006	−2.063	0.070	0.189	0.234
36	−0.518	0.472	−0.008	−0.006	−1.915	0.061	0.192	0.248
37	−0.467	0.489	−0.009	−0.006	−2.024	0.070	0.190	0.234
38	−0.300	0.498	−0.009	−0.006	−2.227	0.068	0.208	0.234
39	−0.448	0.491	−0.009	−0.006	−2.056	0.072	0.186	0.231
40	−0.428	0.489	−0.009	−0.006	−2.100	0.073	0.189	0.228
41	−0.457	0.501	−0.009	−0.006	−1.934	0.063	0.187	0.249
42	−0.132	0.427	−0.009	−0.006	−1.919	0.071	0.078	0.294
43	−0.263	0.494	−0.009	−0.006	−2.200	0.074	0.174	0.237
44	−1.171	0.516	−0.008	−0.005	−1.817	0.103	0.218	0.147
45	−0.313	0.451	−0.009	−0.006	−2.074	0.061	0.184	0.257
46	−0.796	0.352	−0.005	−0.005	−1.806	0.067	0.190	0.224
47	−0.658	0.372	−0.005	−0.005	−1.790	0.054	0.189	0.252
48	−1.638	0.250	−0.001	−0.004	−1.598	0.107	0.207	0.126
49	−0.238	0.491	−0.009	−0.006	−2.123	0.057	0.191	0.259
50	−0.399	0.493	−0.009	−0.006	−2.205	0.079	0.191	0.217
51	−1.866	0.451	−0.007	−0.005	−2.393	0.235	0.170	−0.081
52	−1.771	0.446	−0.007	−0.005	−2.531	0.242	0.163	−0.088
53	−0.439	0.481	−0.009	−0.006	−2.037	0.071	0.180	0.236
54	−0.510	0.486	−0.009	−0.006	−2.101	0.078	0.201	0.213
55	−0.486	0.484	−0.009	−0.006	−2.046	0.070	0.195	0.230
56	1.240	0.405	−0.012	−0.007	−2.528	0.023	0.037	0.431
57	−0.461	0.490	−0.009	−0.006	−2.026	0.069	0.191	0.235
58	−0.105	0.482	−0.008	−0.006	−2.505	0.107	0.123	0.198
59	−0.682	0.455	−0.007	−0.006	−2.459	0.184	0.039	0.086
60	−1.449	0.314	0.006	0.0004	−2.470	0.218	0.024	−0.007
61	−2.266	0.251	0.004	−0.00003	−1.704	0.236	−0.003	−0.028
62	−0.498	0.349	0.001	−0.002	−2.687	0.174	0.004	0.107

**Fig 3:**
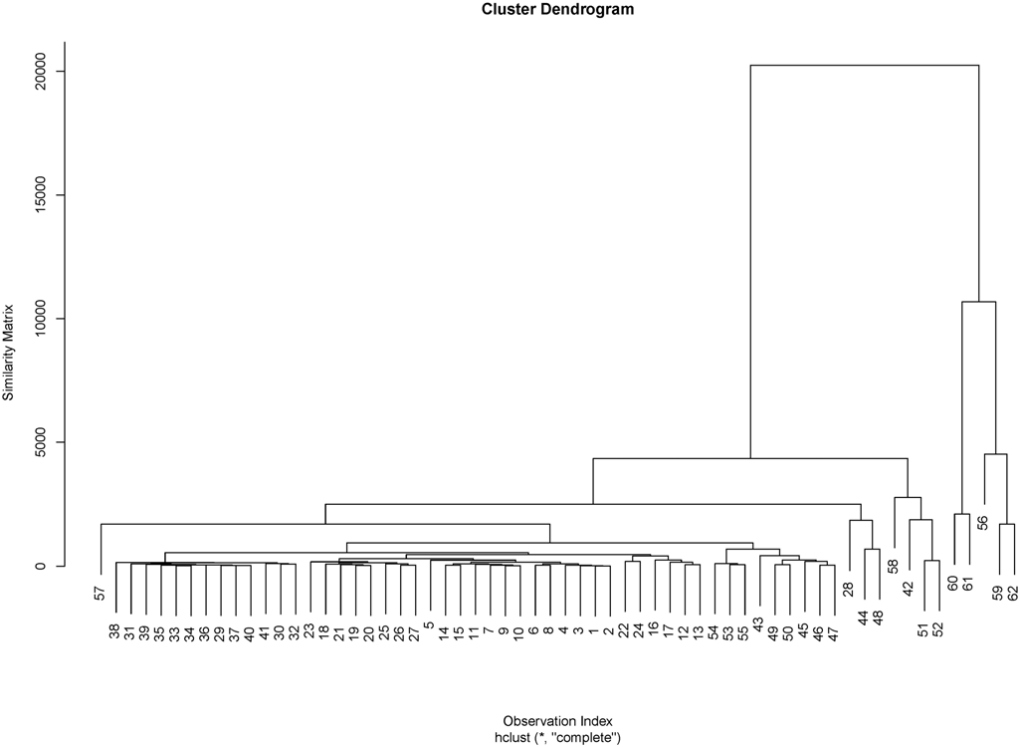
Dendrogram Plot of Pulp Fiber Data.

**Step 5**: The (*n* × *n*) similarity matrix computed for the data in Table 13, using Eq (13) is used to perform an agglomerative hierarchical cluster analysis which yields the dendrogram plot in Fig 3 below.

Notice from Fig 3 that the main cluster *C*_*m*_ contains observations indexed 1–21, 23–27, 29–41, and 43–45. The remaining observations belongs to the *C*_*τi*_ such that *C*_*τ*1_ = {22}, *C*_*τ*2_ = {28}, *C*_*τ*3_ = {42} *C*_*τ*4_ = {46, 47}, *C*_*τ*5_ = {48}, *C*_*τ*6_ = {49, 50}, *C*_*τ*7_ = {51, 52, 56}, *C*_*τ*8_ = {53, 54, 55, 57}, *C*_*τ*9_ = {58}, *C*_*τ*10_ = {59, 62}, *C*_*τ*11_ = {60, 61}

**Step 6**: An *OLS* fit to the data points in the main cluster yields the estimates **b**_*j*_ = -2.0016, 0.5229, -0.0129, -0.0081, -1.2572, 0.1118, 0.3068, 0.0882 and the corresponding prediction variance computed using Eq (14) is $\sigma_{\hat{y}}^{2}=0.0768$. The 11 minor clusters are investigated for likely activation through the *DFFITS*_*τi*_. Their corresponding *DFFITS*_*τi*_ as well as the cutoff value and activation status are

$$\begin{array}{c} \left(\begin{array}{cccc} Minor\;Clusters & DFFITS & \chi_{0.975,8}^{2} & Activation\;Status\\ C_{\tau 1} & 133.416 & 17.535 & Inactive\\ C_{\tau 2} & 14.707 & 17.535 & Activated\\ C_{\tau 3} & 5.135 & 17.535 & Activated\\ C_{\tau 4} & 36.189 & 17.535 & Inactive\\ C_{\tau 5} & 63.314 & 17.535 & Inactive\\ C_{\tau 6} & 104.722 & 17.535 & Inactive\\ C_{\tau 7} & 72.418 & 17.535 & Inactive\\ C_{\tau 8} & 44.316 & 17.535 & Activated\\ C_{\tau 9} & 94.014 & 17.535 & Inactive\\ C_{\tau 10} & 58.145 & 17.535 & Inactive\\ C_{\tau 11} & 80.891 & 17.535 & Inactive \end{array}\right) \end{array}$$ (34)

Notice that the DFIITS-statistics for the second, third and eighth clusters are less than the cutoff value. Hence they are activated in the estimation of *crcc* charting parameters while the other minor clusters remain inactive. The inactive observations are then removed from the pulp fiber dataset **X**. The resulting observation ${\text{x}}_{ij}^{a}$ is then used to compute the mean vector and covariance matrix.

**Step 7**: Having activated the 3 minor clusters, the *crcc* control chart parameters are computed thus:

$$\begin{array}{c} m_{x(crcc)}=(\begin{array}{cccccccc} 0.0070 & 39.8020 & 28.3031 & 1.0633 & 21.8828 & 7.1939 & 5.6620 & 1.0751 \end{array}) \end{array}$$ (35)

and

$$\begin{array}{c} {\hat{\Sigma }}_{\left(crcc\right)}=(\begin{array}{cccccccc} 0.0206 & 1.1654 & -1.1164 & 0.0025 & 0.2690 & 0.0659 & 0.1365 & 0.0642\\ 1.1654 & 84.6795 & -81.8164 & 0.1837 & 21.2941 & 5.4477 & 10.8072 & 4.9675\\ -1.1164 & -81.8164 & 105.6554 & -0.1539 & -15.9059 & -3.9307 & -8.0268 & -3.8934\\ 0.0025 & 0.1837 & -0.1539 & 0.0005 & 0.0575 & 0.0149 & 0.0291 & 0.0131\\ 0.2690 & 21.2941 & -15.9059 & 0.0575 & 7.4543 & 1.9591 & 3.7756 & 1.6970\\ 0.0659 & 5.4477 & -3.9307 & 0.0149 & 1.9591 & 0.5235 & 0.9934 & 0.4466\\ 0.1365 & 10.8072 & -8.0268 & 0.0291 & 3.7756 & 0.9934 & 1.9139 & 0.8597\\ 0.0642 & 4.9675 & -3.8934 & 0.0131 & 1.6970 & 0.4466 & 0.8597 & 0.3913 \end{array}). \end{array}$$ (36)

**Fig 4:**
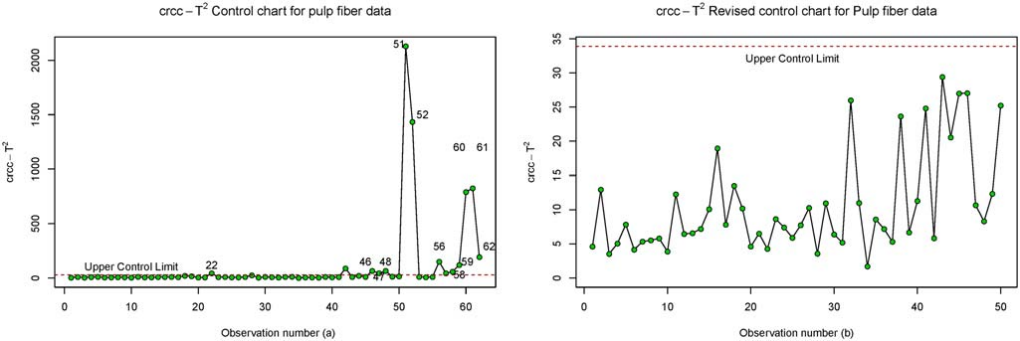
(a) Control Chart for Pulp fiber Dataset. (b) Revised control Chart for Pulp fiber Dataset.

The resulting control chart statistic, $T_{crcc,i}^{2}$ is presented in Table 14 below while the *crcc* control chart is plotted in Fig 4(a).

**Table 14:** The crcc *T* ^2^ statistics.

id	$T_{crcc,i}^{2}$	id	$T_{crcc,i}^{2}$
1	3.695	32	5.163
2	9.212	33	12.425
3	3.613	34	11.286
4	7.715	35	2.391
5	9.512	36	5.382
6	4.686	37	5.841
7	7.255	38	3.784
8	6.407	39	10.972
9	5.568	40	6.392
10	3.642	41	8.461
11	11.788	42	13.646
12	5.070	43	10.647
13	5.857	44	21.743
14	7.556	45	11.313
15	7.432	46	76.696
16	9.916	47	55.086
17	7.354	48	78.127
18	14.002	49	24.348
19	27.804	50	39.697
20	7.945	51	2,136.728
21	7.732	52	1,488.207
22	42.102	53	28.310
23	9.190	54	13.210
24	8.924	55	23.791
25	8.658	56	219.398
26	6.795	57	38.598
27	8.781	58	62.053
28	29.251	59	133.811
29	4.635	60	1,191.909
30	12.527	61	1,187.378
31	7.268	62	292.603

Notice from Table 14 and Fig 4(a) that that observations number 22, 46–48, 51–52, 56, and 58–62 are out-of-control points. According to Rousseeuw et al. [31] and Jung [32], these out-of-control points are said to be observations produced from different woods and different pulping process from those of other observations. The details of these out-of-control points are given below: From the source of the data, it was found that all but the last four pulp samples (observations 59–62) were produced from fir wood. Furthermore, most of the out-of-control samples were obtained using different pulping processes. For instance, observation 62 is the only sample from a chemithermomechanical pulping process, observations 60 and 61 are the only samples from a solvent pulping process, and observations 51, 52, and 56 are obtained from a kraft pulping process. Finally, the smaller outliers (22, 46–48, and 58) all were Douglas fir samples. Consequently, these out-of-control points are removed from the dataset and the *crcc* revised control chart are constructed for the remaining observation. Thus the in-control parameters (mean vector and covariance matrix) computed using algorithm 1 in section 1 are:

$$\begin{array}{c} m_{x(crcc)}=(\begin{array}{cccccccc} 0.0149 & 40.3858 & 27.0086 & 1.0651 & 22.0117 & 7.2208 & 5.7256 & 1.1060 \end{array}) \end{array}$$ (37)

and

$$\begin{array}{c} {\hat{\Sigma }}_{\left(crcc\right)}=(\begin{array}{cccccccc} 0.0235 & 1.3021 & -1.3606 & 0.0030 & 0.3150 & 0.0795 & 0.1593 & 0.0755\\ 1.3021 & 90.3502 & -94.5731 & 0.2069 & 23.0851 & 5.9655 & 11.6883 & 5.4212\\ -1.3606 & -94.5731 & 130.9086 & -0.2010 & -19.7219 & -4.9285 & -9.9184 & -4.8194\\ 0.0030 & 0.2069 & -0.2010 & 0.0006 & 0.0637 & 0.0167 & 0.0322 & 0.0147\\ 0.3150 & 23.0851 & -19.7219 & 0.0637 & 7.8984 & 2.0918 & 3.9938 & 1.8142\\ 0.0795 & 5.9655 & -4.9285 & 0.0167 & 2.0918 & 0.5646 & 1.0588 & 0.4815\\ 0.1593 & 11.6883 & -9.9184 & 0.0322 & 3.9938 & 1.0588 & 2.0209 & 0.9174\\ 0.0755 & 5.4212 & -4.8194 & 0.0147 & 1.8142 & 0.4815 & 0.9174 & 0.4216 \end{array}). \end{array}$$ (38)

while the revised control chart statistic, $T_{crcc-revised,i}^{2}$ is presented in Table 15

**Table 15:** The crcc *T* ^2^ Revised statistics.

id	$T_{rev}^{2}$	id	$T_{rev}^{2}$
1	3.758	26	8.639
2	9.668	27	24.345
3	3.327	28	4.192
4	7.092	29	10.464
5	10.035	30	7.521
6	4.415	31	5.100
7	5.327	32	11.379
8	5.831	33	11.775
9	5.642	34	1.482
10	3.884	35	5.646
11	11.476	36	5.907
12	5.487	37	3.725
13	6.213	38	10.026
14	7.735	39	6.492
15	7.855	40	9.047
16	10.499	41	8.672
17	7.887	42	9.142
18	14.129	43	19.838
19	15.714	44	10.615
20	5.284	45	18.728
21	6.369	46	25.353
22	7.931	47	13.497
23	9.310	48	8.182
24	7.416	49	12.614
25	6.268	50	23.751

The *crcc* control chart is presented in Fig 4(a) while revised control chart based on the in-control-parameters is presented in Fig 4(b). Notice that the process is in a state of statistical control and hence, this in-control parameters can be adopted as a standard for the pulping process and paper produced from them.

The *RMVE* and *RMCD* control charts were also used to analyze the pulp fibre dataset using the function *cov*.*rob* in the *MASS* *package* of *R* software and we found that the results obtained were the same as the proposed method and hence its was not reported.

## Conclusion

The real life application of quality management requires simultaneous monitoring of multiple quality characteristics. Real life data from production and service processes often contains spurious observations whose causes can be traced to assignable variation. This spurious variables often go unnoticed if proper statistical techniques are not employed prior to control chart construction. A multivariate control chart method known as the cluster-regression control chart *crcc* is proposed to simultaneously screen dataset for likely outlier structure and mimic the data trend prior to the construction of the control chart.

Most often, the assumptions needed for large sample theory are better approximated by the distribution of the untrimmed data than by the entire data set, and it is often suggested that statistical analysis should be conducted on the “cleaned data set” when the outliers have been deleted. The proposed method follow this prescription with our objective being that, the parameters of the control chart can be better estimated when the outlying observations which depart from the trend exhibited by the bulk of the dataset have been removed. The proposed method has the tendency to identify mean shift and outliers in datasets while keeping masking and swamping under control.

No single robust algorithm estimator seems to be outstanding, and for any given estimator, it is easier to find outlier configurations and mean shift in datasets where the estimator fails and hence, we state that the performance of the proposed *crcc* method can be quite poor when the level of data contamination go beyond 40% of the sample size.
